# Human Temperaments. IX.—The Envious Temperament


**Published:** 1916-08-19

**Authors:** 


### HUMAN TEMPERAMENTS.

### IX.—The Envious Temperament.*

Wrath is cruel, and anger is outrageous; but who is able to stand before envy?—*Prov.* xxvii. 4.

Envy and jealousy are often used interchangeably, as if they meant the same thing. In fact they are profoundly different. Envy regards one person only, and one person directly. Jealousy regards the relation between two other people. Envy is felt towards him or her who is more fortunate than the envious; jealousy towards him or her who is preferred by some third person to the jealous. In jealousy three people are concerned; in envy only two. Envy and jealousy often go together, but they do not necessarily go together, either in the sense that they are felt towards the same person or in the sense that they are felt by the same person. The envious person is not necessarily a jealous person, nor is the jealous person necessarily envious; but nevertheless the two temperaments often go together.

In the Litany we pray to be delivered from envy, hatred, and malice, and all uncharitableness; but evidently the form of the prayer leaves it doubtful whether we pray to be saved from experiencing these passions or whether we pray to be saved from being the object of them. Either fate is undesirable, and we may well pray to be saved from both. In experience we find that hatred, malice, and uncharitableness are included in envy—included, not as species are included in a genus, but as ingredients are included in a pudding. They are parts of its composition. Envy is, in fact, that hatred, malice, and uncharitableness that is cherished towards those who are more fortunate than the envious. The envious poor man hates the man who is richer than himself; the envious plain woman hates the woman who is better looking.

### The Basis of Envy.

People are not usually envied for what they have achieved or earned. They are envied for that which the caprice of fortune has bestowed upon them. If a man is envied for his achievement, he is envied, not for achieving it, but for the opportunity which came in his way. The more of merit that has gone to procure for him the good things of life, the less he is envied for them. It is the unearned, unmerited good fortune for which he is envied. The envier is outraged by a sense of injustice. Why should unmerited, unearned good fortune come to another rather than to him! He is not absolutely worse off for the good fortune of another, but he is relatively worse off; and the sense of injury rankles in his mind. The natural effect of injury is to provoke retaliation; and since the person who feels himself injured by the good fortune of another cannot retaliate upon the good fortune, or upon the chapter of accidents that bestows the good fortune, he seeks to retaliate upon the recipient of the good fortune. The envious man hates the envied, and "hates anyone the man he would not injure?" The envied man is always in jeopardy from his envier. The retaliation usually takes the form of detraction. Commonly the very fact that the envied is more favoured by fortune than the envier lifts him out of the reach of more serious forms of injury, and detraction is the only weapon left to the envier; but envy has this curious peculiarity, that it is apt to express itself not only—perhaps but slightly—in attacks upon the envied, but also, and more, in attacks on innocent third parties. The envious person is consumed by a rage of hatred against the envied, and if the envied is out of his reach, he will strike blindly against anyone who happens to be within his reach. His unfortunate family are his chief victims. Some piece of good fortune has befallen one of his acquaintances, from whom he must, in spite of himself, conceal his envy, and whom, perhaps, the laws of good breeding compel him, in spite of himself, to congratulate; and the suppression of his passion weights the safety-valve and increases the internal pressure to bursting-point. As soon as he finds himself where he can safely blow off steam, he lets himself go, and then woe to his unfortunate subordinates and dependants! They are amazed by an exhibition of temper for which they have given no provocation, and the source of which is unknown to them.

### The Borderline of Sanity.

If the envious person has no dependants, he, or more often she, for this mode of expressing envy is more frequent in women—is apt to sulk, and the sulkiness is sometimes pushed to such surprising extremes as to raise doubts of the sanity of the sulker. Her family are astounded when the sulker, without rhyme or reason known to them, will refuse to speak, will refuse to eat, will lock herself into her bedroom for days together, will go without fire in the depth of winter, will mortify herself and render the whole household uncomfortable, to all appearance from mere caprice, and without provocation or justification of any kind. Such conduct is scarcely sane—it seems so motiveless. But if we could dive into the mind of the actress, if we could extract a candid and complete confession from her, we should find that she had a motive, and that the motive is a frenzy of envy. Such women or girls are usually obstinately dumb during these attacks, or if they break silence it is only to utter some viperish vituperation; but if we are vigilant, we shall often notice a chance word which gives the instructed mind a clue, and reveals a consuming, raging envy as the underlying motive of the outbreak.

* The previous articles appeared on May 6, 13, 27, June 10 and 24, July 8 and 22, and August 5, 1916; and a letter on the subject on May 20.

### Bacon's Views of Envy.

"A man that is busy and inquisitive is commonly envious," so says Bacon. By "busy" he meant busy in a sense now obsolete, or rather in the sense of "busies himself"—that is to say, a meddler. Bacon's wisdom is much over-rated. He is one of those men who, like Plato, Aristotle, and Mill, have reputations out of all proportion to their deserts. Here he puts the cart before the horse. It is not that meddling and inquisitive people are commonly envious, but that envious people are commonly meddling and inquisitive. Their consciousness of their own merits is a raw surface, which is scarified by having the merits of others brought to their notice; and yet they cannot help tormenting themselves by meddling and inquisitive investigation into the business of other people. In as far as these inquiries discover the defects and misfortunes of other people, in so far the meddler is a happy man or woman; but, alas! his investigations may bring to light only the merits, or the unmerited good fortune, of their subject, and then the envier writhes in torment. But he does not desist. He still hopes to find something to give him consolation, so he still meddles and inquires. Bacon is much nearer the truth when he says "Neither can he that mindeth but his own business find much matter for envy." Here he is unquestionably right. Envy arises from the contemplation of other people's fortunes, and the man who confines his attention to his own business is precluded from envy. "Men of noble birth," he says, "are noted to be envious towards new men when they rise. For the distance is altered; and it is like a deceit of the eye that, when others come on, they think themselves go back." In this he gets near the truth of the matter, that envy arises out of comparison. It is excited by the comparison of the better fortune of others with our own worse fortune. "Lastly," he says, "near kinsfolk and fellows in office, and those that are bred together, are more apt to envy their equals when they are raised." This is true, but Bacon does not see why it is true. Such cases are peculiarly provocative of envy because there is no ignoring them. They thrust themselves upon our notice, and must be attended to. And, moreover, the comparison is more insistent. We are not compelled to compare ourselves with those who are far from us, who are not personally known to us, and with whom we have had little to do; but we cannot help comparing ourselves and our fortunes with those immediately around us, and it is as true of envy as of hatred, of love, and of other personal passions, that frequent personal intercourse is necessary for their development in full strength. However bitterly we may hate a man, the hatred dies away if he goes to India and we never more hear of him. That absence makes the heart grow fonder is only true when the absence is brief.

### A Distinction from Hatred.

Envy includes hatred, but it is not the same as hatred. It is a conditioned hatred, and the hatred lasts only as long as the conditions last—that is to say, as long as the fortune of the envied continues to be better than that of the envier. The hated man is still hated even in misfortune; but let the envied man meet with disaster, and he is no longer an object of hatred. The hatred was conditional, and now the condition is abolished, the hatred goes with it. The envier is quite capable of showing kindness, nay, he finds a genuine delight in showing kindness to the man in his misfortune whom he envied in his good fortune. Envy is the result of comparison, and the result of the comparison is now favourable to the envier, so that he envies no longer; and so delighted is he at the relief to his feelings which the new situation brings, that he is quite capable of active benevolence. Indeed, envy is quite consistent with benevolence and beneficence towards any object that does not inspire envy; and the farther removed the object is from inspiring envy, the more unmistakably and decidedly worse off the person contemplated, the more active and the more genuine the benevolence of the man who bears nothing but ill-will towards those who are better off than himself.

